# Baricitinib for anti-melanoma differentiation-associated protein 5 antibody-positive dermatomyositis-associated interstitial lung disease: a case series and literature review on Janus kinase inhibitors for the disease

**DOI:** 10.1007/s00296-024-05551-2

**Published:** 2024-03-08

**Authors:** Hiroaki Harada, Hirofumi Shoda, Haruka Tsuchiya, Makoto Misaki, Takayuki Sawada, Keishi Fujio

**Affiliations:** Department of Allergy and Rheumatology, Graduate School of Medicine, University of Tokyo, 7-3-1 Hongo, Bunkyo-ku, Tokyo, 113-8655 Japan

**Keywords:** Anti-MDA5 antibody-positive dermatomyositis, Interstitial lung disease, JAK inhibitors, Tofacitinib, Baricitinib

## Abstract

Anti-melanoma differentiation-associated protein 5 antibody-positive dermatomyositis (anti-MDA5-DM) is frequently complicated by progressive interstitial lung disease (ILD), the prognosis of which is poor, and management is a major challenge. We treated three patients with anti-MDA5-DM-associated ILD (anti-MDA5-DM-ILD) using the Janus kinase (JAK) inhibitor, baricitinib, which improved lung opacities and saved two patients. We reviewed 6 patients with anti-MDA5-DM-ILD who had been treated with tofacitinib at our institution. Five of the patients survived, although discontinuation of tofacitinib due to complications was frequently observed. In addition, a literature search of patients with anti-MDA5-DM-ILD who were treated with JAK inhibitors yielded 21 articles involving 79 cases. All patients except one were treated with tofacitinib, and the survival rate was 75.9%. Although not statistically confirmed, the deceased patients tended to be older and had higher ferritin levels. A total of 92 complications were observed, 11 of which resulted in JAK inhibitor discontinuation. Cytomegalovirus reactivation comprised a substantial percentage of all complications and of those patients who required JAK inhibitor discontinuation. Five cases with fatal infective complications were also observed. While tofacitinib has been proposed to be a therapeutic option for anti-MDA5-DM-ILD, other JAK inhibitors, including baricitinib, are a treatment option. Further investigation is warranted to optimize treatment of anti-MDA5-DM-ILD.

## Introduction

Anti-melanoma differentiation-associated protein 5 antibody-positive dermatomyositis (anti-MDA5-DM), which often presents as clinically amyopathic dermatomyositis (CADM), is associated with progressive ILD and has a poor prognosis [1]. Although combined immunosuppressive therapy with glucocorticoids (GCs), cyclophosphamide (CY), and calcineurin inhibitors (CNIs), as well as addition of rituximab (RTX) or plasma exchange (PE) has improved the outcome [2–4], there still remain refractory cases.

Janus kinase (JAK) inhibitors have revolutionized the treatment of inflammatory diseases. Tofacitinib has become a promising agent for anti-MDA5-DM-associated ILD (anti-MDA5-DM-ILD), but reports involving other JAK inhibitors are extremely rare [5, 6]. We recently treated three anti-MDA5-DM-ILD patients with baricitinib. To the best of our knowledge, they are the first reported cases of anti-MDA5-DM-ILD treated with baricitinib. Herein, we describe these cases, and review other anti-MDA5-DM-ILD patients treated with tofacitinib at our institution and reports of anti-MDA5-DM-ILD patients treated with JAK inhibitors, focusing on efficacy and safety.

## Case descriptions

**Fig. 1:**
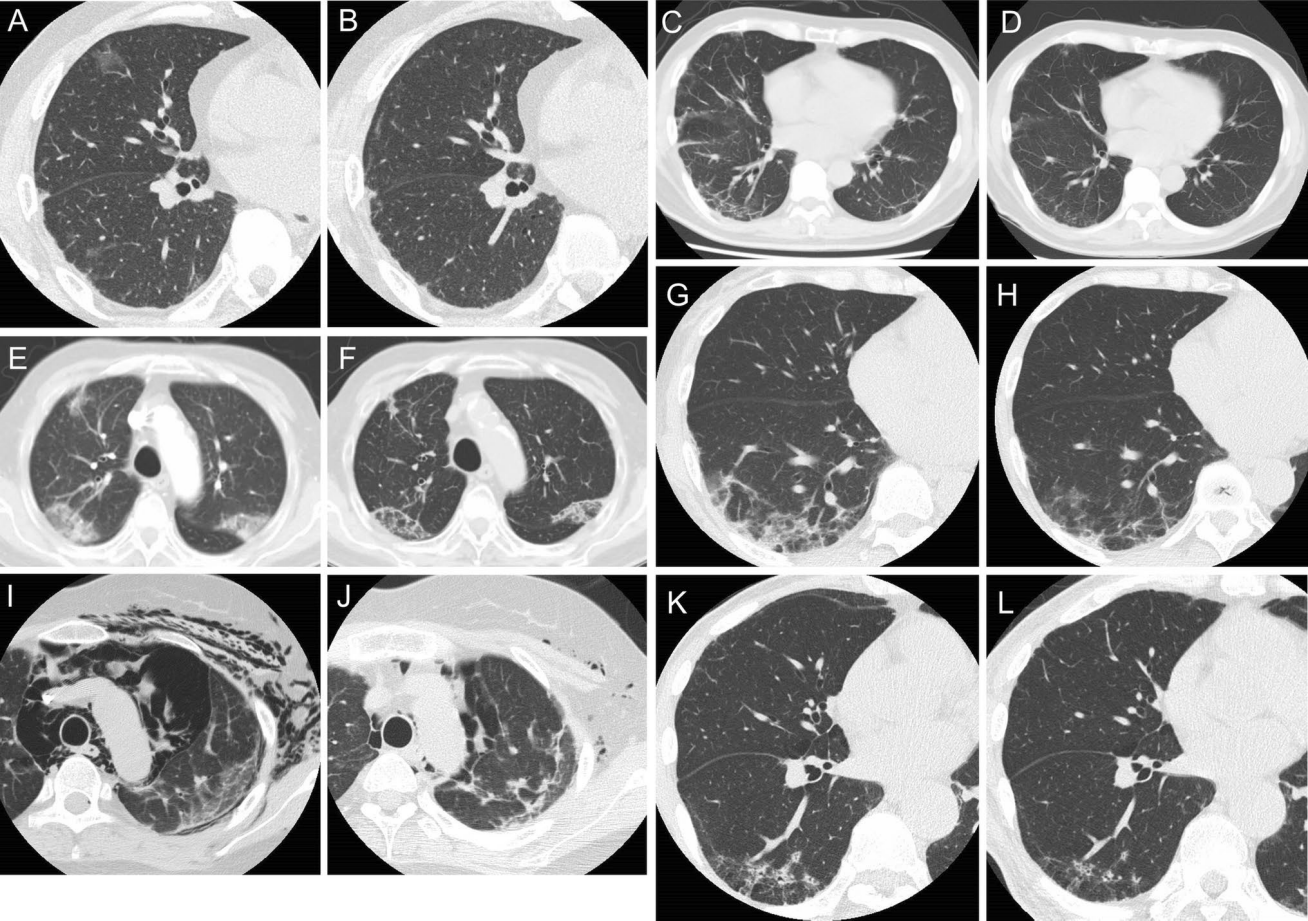
Chest computed tomography imaging. Case 1: **A** before and **B** 2 weeks after baricitinib initiation; Case 2: **C** before and **D** 5 days after baricitinib initiation; Case 3: **E** before and **F** 4 weeks after baricitinib initiation; Case 4: **G** before and **H** 4 weeks after tofacitinib initiation; Case 5: **I** before and **J** 5 weeks after tofacitinib initiation; Case 9: **K** before and **L** 5 weeks after tofacitinib initiation

**Fig. 2:**
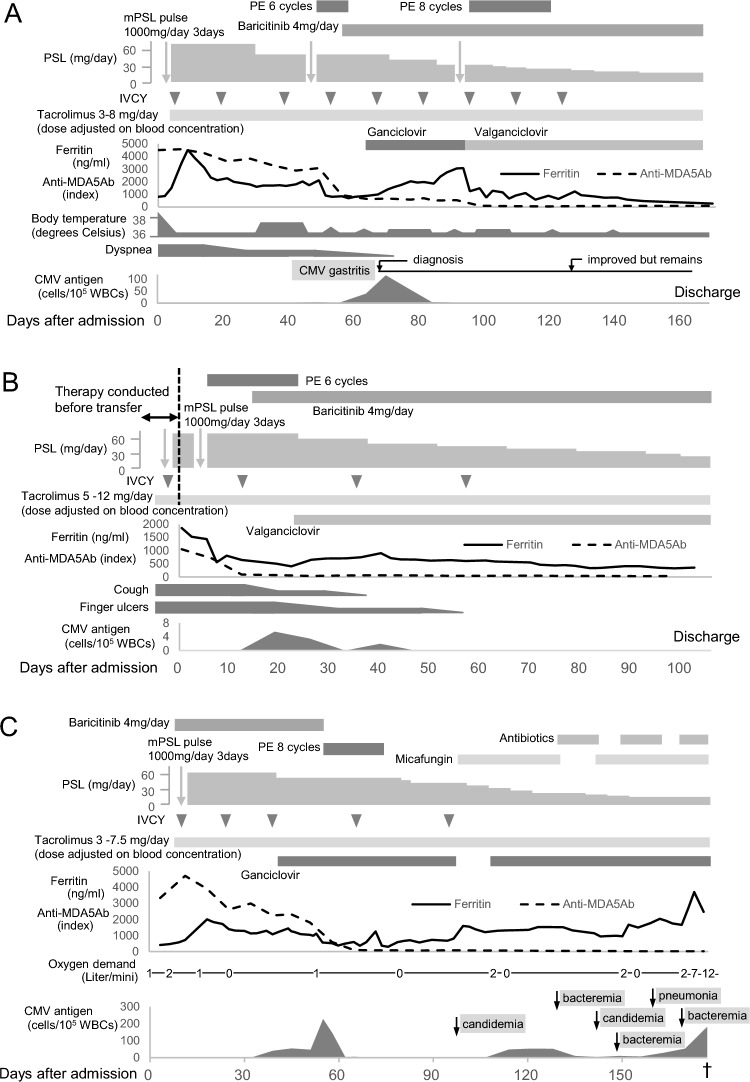
Clinical course of cases treated with baricitinib. Case 1: A; Case 2: B; Case 3: C. Anti-MDA5Ab, anti-melanoma differentiation-associated protein 5 antibody; *CMV* cytomegalovirus; *IVCY* intravenous cyclophosphamide; *mPSL* methylprednisolone; *PE* plasma exchange; *PSL* prednisolone; *WBCs* white blood cells

- Case 1: A 55-year-old male was hospitalized with complaints of fevers, skin eruptions, gait disturbance, dysphasia, and dyspnea which had developed in the last 10 days. Gottron’s papules were seen on his fingers, weakness of proximal muscles was found, chest computed tomography (CT) showed bilateral lung opacities, and anti-MDA5 antibody was positive. Under the diagnosis of anti-MDA5-DM-ILD, combination therapy with high-dose GCs, CY, tacrolimus was initiated. Although he got improved transiently, fever relapsed and lung infiltrates had become enlarged on CT (Fig. 1A). Addition of pulse glucocorticoid and PE had limited efficacy. After initiation of baricitinib 4 mg/day, slight improvement of ILD was achieved (Fig. 1B). Although gastric ulcers with a CMV infection (diagnosed by immunohistochemical CMV detection on biopsy specimens) and venous thrombophlebitis developed, they were controlled by antiviral therapy with ganciclovir and anticoagulation, respectively. Five months elapsed before ILD was stabilized, at which time he was discharged on combination treatment with baricitinib. His clinical course was summarized in Fig. 2A.
- Case 2: A 57-year-old male diagnosed with anti-MDA5-DM-ILD was transferred to our hospital for alternative treatment because combination therapy with high-dose GC, CY, and tacrolimus was ineffective. Although he had no fever or dyspnea, he suffered from persistent cough and digital ulcers. Hyperferritinemia (3529 ng/ml at the initiation of therapy and 1855 ng/ml on admission) was also noted. Baricitinib and PE were initiated, which improved ILD (Fig. 1C, D) as well as cough and digital ulcers. Although CMV reactivation (blood CMV antigen detected by phosphoprotein 65 staining was positive: 5.5 per 10^5^ white blood cells (WBCs)) and intestinal pneumatosis developed and required antiviral therapy with valganciclovir and bowel rest, respectively, ILD remained stable on combination treatment with baricitinib (4 mg/d). His clinical course was summarized in Fig. 2B.
- Case 3: A 79-year-old male with seronegative rheumatoid arthritis was hospitalized for fever and dyspnea. Antibiotics administration assuming bacterial pneumonia was ineffective. Since he had facial erythema that was pathologically characterized by perivascular dermatitis with mucin deposition and anti-MDA5 antibody was positive, he was diagnosed with severe CADM-associated ILD with oxygen demand. Treatment with baricitinib, in addition to immunosuppressive therapy with GCs, CY, and tacrolimus, gradually improved the ILD (Fig. 1E, F). However, we were compelled to discontinue baricitinib due to CMV reactivation (blood CMV antigen per 10^5^ WBCs was 228 at maximum). Although it was initially resistant to ganciclovir administration, it gradually subsided after baricitinib withdrawal. Thereafter, ILD followed a smoldering course, and the patient succumbed to successive infections after 6 months of treatment. His clinical course was summarized in Fig. 2C.

## Methods

### Review of past cases who had been treated with tofacitinib at our institution

We conducted a retrospective chart review of anti-MDA5-DM-ILD patients treated with tofacitinib in the Department of Allergy and Rheumatology at the University of Tokyo Hospital from January 2017 to June 2023. The following data were collected: age; gender; diagnosis (DM or CADM); laboratory parameters; imaging findings; co-existing autoimmune diseases; indication and response of tofacitinib; concomitant therapy; complications; and outcomes. These review processes were in accordance with the ethical standards of the Helsinki Declaration and were approved by the Research Ethics Committee of the University of Tokyo (number 2431).

### Literature review

We reviewed the literature relevant to treatment of anti-MDA5-DM-ILD with JAK inhibitors. We searched the PubMed database on 8 January 2024 using the following keywords or medical subject heading (MeSH) terms: (MDA5 [all fields] OR melanoma differentiation-associated gene 5 [all fields] OR melanoma differentiation-associated protein 5 [all fields]) AND (lung diseases, interstitial [MeSH terms]) AND (Janus kinase inhibitors [MeSH terms] OR tofacitinib [all fields] OR baricitinib [all fields] OR upadacitinib [all fields] OR filgotinib [all fields] OR peficitinib [all fields] OR abrocitinib [all fields] OR brepocitinib [all fields] OR ritlecitinib [all fields] OR ruxolitinib [all fields] OR delgocitinib [all fields] OR fedratinib [all fields] OR momelotinib [all fields] OR pacritinib [all fields] OR itacitinib [all fields] OR decernotinib [all fields] OR deucravacitinib [all fields]). We also searched the Web of Science and Directory of Open Access Journals using the following terms: interstitial lung disease AND (MDA5 OR melanoma differentiation-associated gene 5 OR melanoma differentiation-associated protein 5) AND (Janus kinase inhibitors OR tofacitinib OR baricitinib OR upadacitinib OR filgotinib OR peficitinib OR abrocitinib OR brepocitinib OR ritlecitinib OR ruxolitinib OR delgocitinib OR fedratinib OR momelotinib OR pacritinib OR itacitinib OR decernotinib OR deucravacitinib). Their relevance to the topic was checked by reading the abstract and full text. Review articles and non-English articles were excluded. Juvenile cases were also excluded because they might have different immunological background from the adults, which could possibly confound our analysis. Two articles referenced by the literatures in the initial search were added. The following information was collected: age; gender; diagnosis (DM or CADM); laboratory parameters; co-existing autoimmune diseases; positioning of JAK inhibitors (initiated within 7 days of treatment was considered “initial”, initiated after 7 days of treatment was considered “additional”, and initiated after relapse was considered “re-induction”); concomitant therapy; complications; and outcomes. These parameters were compared between survived and non-survived patients.

### Statistical analysis

The ratios and ordinal variables between the two groups were compared using Fisher’s exact test and the Mann–Whitney *U* test, respectively. All statistical analyses were performed with EZR (Saitama Medical Center, Jichi Medical University, Saitama, Japan), which is a graphical user interface for R (The R Foundation for Statistical Computing, Vienna, Austria) and is a modified version of R commander designed to add statistical functions frequently used in biostatistics [7].

## Results

### Characteristics and outcome of patients treated with JAK inhibitors at our institution

We identified 6 anti-MDA5-DM-ILD patients treated with tofacitinib (cases 4–9). The characteristics and outcomes of the patients treated with baricitinib or tofacitinib are summarized in Table 1. One patient was male, two patients were clinically amyopathic, and the median age was 58.5 years. One patient had a history of cutaneous lupus erythematosus. None of the patients reported a history of varicella zoster vaccination. While all patients received three or more immunosuppressive agents, most of them required tofacitinib for refractory ILD. Another patient switched from previous treatment due to adverse events and the other had disease activity other than ILD. All patients experienced complications, which lead discontinuation of tofacitinib in five cases. Despite these drawbacks, three patients achieved ILD improvement (Fig. 1G–L) and five patients survived.

**Table 1:** Characteristics and treatment outcome of anti-MDA5-DM-associated ILD patients treated with JAK inhibitors at our institution

Jak inhibitor	Baricitinib			Tofacitinib					
Patients	Case 1	Case 2	Case 3	Case 4	Case 5	Case 6	Case 7	Case 8	Case 9
Gender	Male	Male	Male	Female	Female	Female	Female	Female	Male
Age, years	55	57	79	62	21	55	73	45	66
Diagnosis	DM	DM	CADM	CADM	DM	DM	DM	DM	CADM
CK, U/L	1199	634	45	214	220	54	891	311	37
LD, U/L	534	368	247	337	474	257	471	421	570
CRP, mg/dL	0.94	2.16	4.54	0.50	1.87	0.26	3.00	0.30	0.29
Ferritin, ng/mL	808	3529	403	408	429	465	401	1746	1694
KL-6, U/mL,	575	1320	280	1085	575	1719	312	454	458
Accompanied autoimmune diseases	None	None	RA	CLE	None	None	None	None	None
Indication	Treatment resistance	Treatment resistance	Severe disease	Treatment resistance	Treatment resistance	Treatment resistance	Treatment resistance	Switch from previous treatment^a^	Disease activity other than ILD^b^
Concomitant treatment	GC, CNI, CY, PE	GC, CNI, CY, PE	GC, CNI, CY	GC, CNI, CY, PE	GC, CNI, CY, PE	GC, CNI, CY, PE	GC, CNI, CY, PE	GC, CNI, CY	GC, CNI, CY, PE
Treatment response	Yes	Yes	Yes	Yes	Yes	No (unchanged)	No (become worse)	NA^c^	Yes
Complications	CMV gastritis, venous thrombosis	CMV reactivation, intestinal pneumatosis	CMV reactivation	Pulmonary embolism, venous thrombosis	Bacteremia, BK viral nephropathy	Cytopenia	CMV reactivation, cytopenia, angina, pneumothorax, pneumomediastinum	CMV reactivation	CMV reactivation, liver injury
Discontinuation due to complications	No	No	Yes	No	Yes	Yes	No	Yes	Yes
Outcome	Survived	Survived	Deceased	Survived	Survived	Survived	Deceased	Survived	Survived

*anti-MDA5-DM* anti-melanoma differentiation-associated protein 5 antibody-positive dermatomyositis; *CADM* clinically amyopathic dermatomyositis; *CK* creatinine kinase; *CLE* cutaneous lupus erythematosus; *CMV* Cytomegalovirus; *CNI* calcineurin inhibitors; *CRP* C-reactive protein; *CY* cyclophosphamide; *DM* dermatomyositis; *GC* glucocorticoid; *ILD* interstitial lung disease; *JAK* Janus kinase; *KL-6* Krebs von den Lungen-6; *LD* lactate dehydrogenase; *NA* not assessed; *PE* plasma exchange; *PsA* psoriatic arthritis; *RA* rheumatoid arthritis; *SD* standard deviation

^a^Previous treatment (plasma exchange) was effective but had to be discontinued due to bacteremia possibly related to blood access catheter

^b^Previous treatment improved ILD, but thrombotic microangiopathy developed and tofacitinib and plasma exchange were added

^c^Tofacitinib was discontinued before the assessment of its efficacy

### Literature review

The flowchart of our literature search is shown in Fig. 2. A total of 21 articles, including 79 cases, were identified [5, 6, 8–26]. Although Shirai et al. [8] reported 13 patients treated with tofacitinib, only 8 patients were included in our analysis because data of the other 5 patients were combined with those treated without tofacitinib. Tofacitinib was used in 78 patients and peficitinib was used in the other patient; no other JAK inhibitors were used.

Table 2 shows a summary of the patient characteristics and outcomes in the reviewed articles. The mean age was 52.9 years, 32 patients (40.5%) were male, and 49 patients (69% among 71 patients) were diagnosed with CADM. The mean levels of serum creatine kinase (CK), lactate dehydrogenase, C-reactive protein (CRP), ferritin, and Krebs von den Lungen-6 (KL-6) were 212 U/L, 366 U/L, 1.72 mg/dL, 1304 ng/mL, and 867 U/mL, respectively. Twenty-nine patients were anti-Ro52 antibody-positive. One patient each was diagnosed with rheumatoid arthritis and psoriatic arthritis. All patients except one received GC; 32, 27, 20, 11, and 2 patients received CNI, CY, PE, RTX, and MMF, respectively. Seven patients received intravenous immunoglobulins and 4 patients received pirfenidone. Ninety-two complications were recorded. The most frequent complications were viral infections, followed by cytopenia, fungal infections, and bacterial infections. Among viral infections, CMV reactivation was observed more frequently than herpes zoster. Eleven patients discontinued JAK inhibitors due to complications. At least 3 patients discontinued JAK inhibitors due to CMV reactivation. Fan et al. [11] reported 5 fatalities due to infections (4 pulmonary fungal infections and 1 sepsis) in their retrospective study. Sixty (75.9%) patients survived.

**Table 2:** Characteristics and treatment outcome of anti-MDA5-DM-associated ILD patients treated with JAK inhibitors in the previous literatures

JAK inhibitors (number of patients)	Tofacitinib (78), Peficitinib (1)
Gender, male (%)	32 (40.5)
Age, years, mean (SD)	52.9 (11.5)
Diagnosis (number of patients)	DM (22), CADM (49), NA (8)
CK, U/L, mean (SD), available number of patients	212 (507), 40
LD, U/L, mean (SD), available number of patients	366 (178), 61
CRP, mg/dL, mean (SD), available number of patients	1.72 (2.24), 40
Ferritin, ng/mL, mean (SD), available number of patients	1304 (1438), 48
KL-6, U/mL, median (IQR), available number of patients	867 (501), 37
Accompanied autoimmune diseases (number of patients)	Psoriatic arthritis (1) Rheumatoid arthritis (1)
Positioning (number of patients)	Initial (29), Additional (22), Re-induction (2), NA (26)
Concomitant treatment (number of patients)	GC (78), CNI (32), CY (27), PE (20), RTX (11), IVIG (7), Pirfenidone (4), MMF (2), Unspecified (11)
Complications	Viral infections (38) CMV (23), herpes zoster (7), others (8) Bacterial infections (12) Respiratory (6), sepsis (4), others (2) Fungal infections (14) Other events Cytopenia (15), Pneumomediastinum (3), Thrombotic microangiopathy (2), Renal dysfunction (1), Venous thrombosis (1), Alveolar proteinosis (1), Liver dysfunction (1), Hepatic failure (1), Shock of unknown cause (1), Intramuscular bleeding (1), Hip fracture (1)
Number of discontinuation cases due to complications	11
Number of survivors (%)	60 (75.9)

*CADM* clinically amyopathic dermatomyositis; *CK* creatinine kinase; *CMV* cytomegalovirus; *CNI* calcineurin inhibitors; *CRP* C-reactive protein; *CY* cyclophosphamide; *DM* dermatomyositis; *GC* glucocorticoid; *IQR* interquartile range; *IVIG* intravenous immunoglobulin; *JAK* Janus kinase; *KL-6* Krebs von den Lungen-6; *LD* lactate dehydrogenase; *MMF* mycophenolate mofetil; *NA* not assessed; *PE* plasma exchange; *RTX* rituximab; *SD* standard deviation

**Fig. 3:**
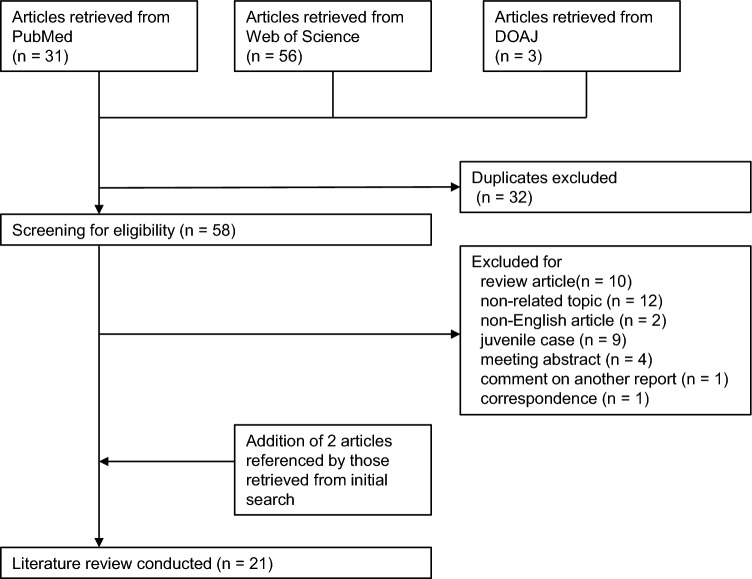
Flowchart of literature search. *DOAJ* Directory of Open Access Journals

We classified the patients into survivors and non-survivors (Table 3). Gender, diagnosis, levels of CK, CRP, and KL-6, tofacitinib positioning, and number of concomitant treatments were comparable between the two groups. Although the other parameters could not be statistically compared because individual data were not provided in some articles, patient age and ferritin appeared to be higher in deceased patients (Fig. 3).

**Table 3:** Comparisons between survived and deceased patients in the previous literatures

	Survived *n* = 60	Deceased *n* = 19	*P* value
JAK inhibitors (number of patients)	Tofacitinib (59) Peficitinib (1)	Tofacitinib (19)	–
Gender, male (%), number of identified patients	20 (39.2), 51	8 (61.5), 13	0.212
Age, years, mean (SD), available number of patients	50.6 (11.5), 45	57.9 (10.8), 8	Not applicable
Diagnosis (number of identified patients)	CADM (29) DM (10)	CADM (3) DM (3)	0.334
CK, U/L, median (IQR), available number of patients	167 (130–404.5), 11	155 (123–341.5), 3	0.769
LD, U/L, mean (SD), available number of patients	362 (229), 31	472 (105), 4	Not applicable
CRP, mg/dL, median (IQR), available number of patients	0.625 (0.295–1.31), 11	1.07 (0.63–3.56), 3	0.633
Ferritin, ng/mL, mean (SD), available number of patients	1143 (1244), 41	2240 (2242), 7	Not applicable
KL-6, U/mL, median (IQR), available number of patients	788.5 (425.75–1104.5), 32	802 (661–913), 5	0.813
Positioning (number of identified patients)	Initial (26) Additional (17) Re-induction (2)	Initial (3) Additional (5) Re-induction (0)	0.474
Number of concomitant treatments per patient, median (IQR), available number of patients	3(2–4), 33	3(2–4), 12	0.916

*CADM* clinically amyopathic dermatomyositis; *CK* creatinine kinase; *CRP* C-reactive protein; *DM* dermatomyositis; *IQR* interquartile range; *JAK* Janus kinase; *KL-6* Krebs von den Lungen-6; *LD* lactate dehydrogenase; *NA* not assessed; *SD* standard deviation

## Discussion

Anti-MDA5-DM frequently complicates refractory progressive ILD, which has a mortality rate as high as 50% despite intensive immunosuppressive therapy [1, 6]. Although the exact mechanism underlying recalcitrance to treatment remains elusive, marked elevation of serum ferritin and cytokines implicates an excessive activation of macrophages [27–30]. Furthermore, evidence suggesting involvement of type I interferon (IFN-I) signaling is accumulating [31, 32]. MDA5, a cytosolic sensor of virus-originated double-stranded RNA that triggers IFN-I signaling, might be released from lysis of virus-infected cells and induce autoantibody production. It is supported by the seasonal and geographic clusters of anti-MDA5-DM [33, 34]. Anti-MDA5 antibodies may participate in the disease process through the formation of immune complex that contains RNA and induces IFN‐α production via Toll‐like receptor 7 [35]. One study reported a strong correlation between the anti-MDA5 antibody titer and the serum CX3CL1 concentration [29]. CX3CL1, which is responsible for recruitment of CX3CR1-positive M2 macrophages, is produced by vascular endothelial cells with IFN-I stimulation [36, 37]. It can be hypothesized that activation of IFN-I signaling, in which anti-MDA5 antibody may have a role, induces lung fibrosis through the accumulation of pathologic M2 macrophages recruited by CX3CL1 in anti-MDA5-DM-ILD [34].

JAK inhibitors exert immunomodulatory action through suppression of intracellular signaling involving JAK proteins. Since JAK proteins are ubiquitously expressed and involved in the signaling of multiple cytokines, there is an expectation of their application in refractory diseases in which redundant inflammatory process might exist. Our cases suggest the therapeutic potential of JAK inhibitors for anti-MDA5-DM-ILD. ILD improved in all 3 patients treated with baricitinib and in 3 out of 6 patients treated with tofacitinib. Although the efficacy of other therapies administered concomitantly or beforehand that appeared with a delay cannot be excluded, improvement was not observed before initiation of JAK inhibitors in most cases. After remission was achieved, two patients treated with baricitinib and one patient treated with tofacitinib remained stable, which further indicates the role of JAK inhibitors in maintaining anti-MDA5-DM-ILD remission.

Among the reviewed studies, almost all patients were treated with tofacitinib and survival rate was 75.9%. Given the recalcitrance of patients treated with JAK inhibitors, the outcomes were favorable, although not satisfactory. A comparison of survived and deceased patients suggested that higher ferritin levels and older age were associated with poor outcomes, which is consistent with the findings of previous studies [5, 38]. However, these parameters were not directly linked to outcome, as observed in cases 3 and 7 (fatal outcomes) despite low levels of ferritin initially. Further investigation is needed to elucidate the prognostic factors for anti-MDA5-DM patients treated with JAK inhibitors.

Although tofacitinib has been predominantly used, it is not known whether tofacitinib is preferable to other JAK inhibitors. Since JAK inhibitor selectivity against JAK proteins varies, the efficacy could be different. Tofacitinib inhibits JAK1, JAK3, and to a lesser extent JAK2, whereas baricitinib inhibits JAK1, JAK2, and to a lesser extent TYK2 [39]. Thus, both agents efficiently suppress IFN-I signaling that utilizes JAK1. Indeed, baricitinib improved the symptoms of interferonopathies [40]. JAK1 inhibition might control anti-MDA5-DM-ILD through abrogating the IFN-I pathway. In contrast, compared to tofacitinib, baricitinib is more potent in inhibiting JAK2. JAK2 mediates transforming growth factor β signaling in fibroblasts [41, 42], and its activation is observed in lung fibrosis [43, 44]. For patients with rheumatoid arthritis-associated ILD, baricitinib treatment for ≥ 6 months reduced fibrotic and inflammatory biomarkers and achieved stabilization or improvement of the disease [45, 46]. Taken together, baricitinib might regulate anti-MDA5-DM-ILD through multiple mechanisms of action. Our observation of baricitinib efficacy warrants further investigation to verify the usage of baricitinib for anti-MDA5-DM-ILD.

It is essential to exercise caution with JAK inhibitors regarding adverse events especially in older or already immunosuppressed patients. Not a few patients had to discontinue JAK inhibitors for adverse events, and fatal infection also occurred. Compared to those reported in the clinical trials for rheumatoid arthritis or other autoimmune diseases, the incidence of CMV reactivation appeared to be high [39], indicating a more profound immunosuppressive state in anti-MDA5-DM-ILD patients. We have to pay more attention to CMV reactivation in light of the resistant cases as observed in case 3 and our previous observation of a high CMV titer linked to poor outcome in immunosuppressed patients with autoimmune diseases [47]. In addition to close monitoring of viral load and relating visceral signs, prompt and judicious action of initiating antiviral therapy and/or withholding JAK inhibitors would be required.

A limitation of our literature review is publication bias. Unfavorable cases are more likely to go unreported, which may lead to overestimation of JAK inhibitor efficacy. Another limitation is the lack of control patients, which prevented us from estimating the effect of JAK inhibitors. Since anti-MDA5-DM-ILD is still a life-threatening disorder, there exists an ethical problem to conduct a control study which limits treatment options, especially for intractable cases. Comparison with historical cases could be helpful, but recent advances in treatment strategies, including RTX or PE, make it difficult to assume which effect would be attributable to JAK inhibitors. A detailed analysis based on more accumulation of cases is necessary to solve the problem.

In conclusion, we treated three cases of anti-MDA5-DM-ILD with baricitinib, which showed potential therapeutic benefit. This is comparable with the findings of past cases treated with tofacitinib, while the discontinuation rate was relatively high. Reports on the efficacy of other JAK inhibitors are lacking, and the significance of inhibiting both JAK1 and JAK2 remains to be elucidated. Further investigations are required to optimize the use of JAK inhibitors, including baricitinib, for the treatment of anti-MDA5-DM-ILD.

## Data availability

The data related to this article would be available from the corresponding author upon reasonable request.
